# Role of Vessel Wall Magnetic Resonance Imaging in the Diagnosis of Intracranial Vasculopathies

**DOI:** 10.7759/cureus.73714

**Published:** 2024-11-14

**Authors:** Kusum L Yadav, Ankita Gupta, Chaturbujh P Swarnkar, Naima Mannan

**Affiliations:** 1 Radiodiagnosis, Sawai Man Singh (SMS) Medical College and Hospital, Jaipur, IND

**Keywords:** arterial remodeling, cns vasculitis, ct and mr angiography, moyamoya disease, vessel wall mri

## Abstract

Background: Stroke is a leading cause of death and disability worldwide, affecting millions annually. Accurate etiological diagnosis is critical for the effective treatment and prevention of recurrent strokes. Traditional luminal imaging techniques like computed tomography (CT) and magnetic resonance angiography (MRA) provide limited information, focusing solely on vessel lumen characteristics. Vessel wall magnetic resonance imaging (VW-MRI) has emerged as a valuable non-invasive technique for evaluating intracranial vasculopathies with high spatial resolution. It also helps in identifying plaque composition and distinguishing between lipid-rich, fibrous, calcified, and thrombotic components. This information is crucial for assessing plaque vulnerability and predicting the risk of future cardiovascular events.

Objectives: This study aimed to evaluate the findings of VW-MRI among cases having suspicious stenosis detected on MRA and to characterize atherosclerotic plaque vulnerability features on VW-MRI.

Methods: This cross-sectional hospital-based study was conducted at the Department of Radiodiagnosis, Sawai Man Singh (SMS) Medical College and Hospital, Jaipur, on 45 patients who met the inclusion criteria. Participants who showed suspicious stenosis on conventional MRA underwent VW-MRI using a 3T GE Signa Architect 64-channel MRI machine (GE HealthCare, Chicago, IL, USA) to obtain vessel wall MR sequences: 3D Ax time-of-flight (TOF) fat-suppressed spoiled gradient recalled echo (SPGR) Fs, 3D Sag T2 Cube, 3D Sag T1 black-blood (BB) Cube Fs, 3D Sag T1 magnetization-prepared rapid gradient-echo(MP-RAGE), and 3D Sag T1 BB Cube Fs +C. These sequences will be taken to differentiate between intracranial atherosclerotic plaque, vasculitis, and moyamoya disease/pattern and also to characterize atherosclerotic plaque vulnerability features. Data were collected through a structured questionnaire and magnetic resonance imaging (MRI) results, followed by statistical analysis.

Results: The majority (50%) of patients were middle-aged, with a mean age of 48 years. Atherosclerosis was the most common (67%) diagnosis, followed by central nervous system (CNS) vasculitis (22%) and moyamoya disease (11%). VW-MRI detected significant VW abnormalities in all cases as compared to MRA which detected only 73.35% of cases. Positive remodeling was associated with CNS vasculitis and vulnerable plaques, while moyamoya disease was linked to negative remodeling.

Conclusion: VW-MRI proves superior to traditional luminal imaging for diagnosing intracranial vasculopathies. It enhances the understanding of VW abnormalities, aiding in the management of stroke and other cerebrovascular diseases.

## Introduction

Cerebrovascular diseases are considered to be among the leading causes of cognitive impairment, debilitation, and even death worldwide. For the diagnosis, we already have an algorithm in place in which we use magnetic resonance angiography (MRA)/computed tomography angiography (CTA)/digital subtraction angiography (DSA) to evaluate intracranial vasculopathy. However, these luminal-based techniques have limited specificity for disease differentiation, specifically if you look at central nervous system (CNS) vasculitis. It's important to distinguish between reversible cerebral vasoconstriction syndrome (RCVS) and vasculitis because they require different treatment approaches. Vasculitis is typically managed with immunosuppressive medications, while RCVS is managed with symptomatic therapy and calcium channel blockers. So, delayed therapy or incorrect therapy can result in significant morbidity and/or mortality. Vessel wall magnetic resonance imaging (VW-MRI) overcomes these limitations, and adding this technology to conventional imaging is likely to be beneficial in many ways like characterizing various conditions like intracranial atherosclerotic plaque, vasculitis, reversible vasoconstriction of intracranial vasculature, and cerebral arterial dissection to diagnose intracranial arterial narrowing. Schaafsma et al. studied 205 stroke/transient ischemic attack (TIA) patients, and VW-MRI revised the cause of stroke for almost half of the patients [1]. A recent systematic review by Wang et al. found that 50.6% of stroke patients with non-stenotic vessels had intracranial plaque, according to VW-MRI. The result comes with a 95% confidence interval [2]. So, the probability of an accurate diagnosis in cases of non-stenotic vasculopathy markedly raised when VW-MRI findings were assessed alongside luminal imaging [3]. Another important feature of VW-MRI is differentiating between these symptomatic and non-symptomatic plaques. There is a presence of enhancement in the symptomatic plaques on post-contrast images. The cause for this enhancement in plaques is attributed to inflammation, neovascularization, and impaired endothelial function [4]; also, the more vivid the plaque enhancement, the higher the likelihood of future stroke events [5]. Vessel remodeling is another indicator of plaque activity. Positive remodeling involves a compensatory bulging of the VW, while negative remodeling indicates fibrosis. Also, the presence of hemorrhage in the plaques, which appear as T1-weighted hyperintense, heightened the risk of stroke [6]. 

Our study aims to describe the characteristic features of various intracranial vasculopathies on VW-MRI. 

## Materials and methods

This hospital-based descriptive study was conducted with the necessary approval from the Institutional Ethics Committee of Sawai Man Singh (SMS) Medical College and Hospital (approval number: 556/MC/EC/2023) and informed consent. A total of 45 patients were included and had undergone VW-MRI using a 3T GE Signa architect 64-channel MRI machine (GE HealthCare, Chicago, IL, USA) from 2021 to 2023, provided they met the specified inclusion and exclusion criteria. VW-MRI sequences were employed, such as 3D Ax time-of-flight (TOF) fat-suppressed spoiled gradient recalled echo (SPGR) Fs, 3D Sag T2 Cube, 3D Sag T1 black-blood (BB) Cube Fs, 3D Sag T1 magnetization-prepared rapid gradient-echo (MP-RAGE), and 3D Sag T1 BB Cube Fs +C, and data is analyzed using images from these sequences. These sequences allowed for the differentiation of intracranial atherosclerotic plaques, vasculitis, and moyamoya patterns, as well as the characterization of plaque vulnerability features. Non-vulnerable atherosclerosis is characterized by arterial wall thickening, luminal narrowing, irregular inner margins, and eccentric post-contrast enhancement. CNS vasculitis is characterized by circumferential wall enhancement and regular inner margins, differentiating it from atherosclerotic conditions. Moyamoya disease is distinguished by the absence of post-contrast enhancement and the presence of significant luminal narrowing without hemorrhagic components. Plaque enhancement on T1-weighted post-contrast sequences is a characteristic of symptomatic plaque and reflects intralesional inflammation and neovascularization.

Inclusion criteria

Participants included adults aged 18 years and older who had experienced a stroke within two weeks of onset, as detected by MRI, and who exhibited suspicious lesions on MRA.

Exclusion criteria

Exclusion criteria included patients with ferromagnetic implants, pacemakers, aneurysm clips, or other contraindications to MRI. Patients with claustrophobia, those who refused consent for VW-MRI, and those with incidental findings like brain tumors or arteriovenous malformations (AVM) were also excluded. 

## Results

The study included 45 patients, with the majority (50%) aged between 51 and 60 years and a mean age of 48±14.1 years. Males (53%) and females (47%) were most represented in this age group. Regarding comorbidities, 64% had hypertension, 56% had diabetes, and 29% had ischemic heart disease. Smoking was reported in 33% of patients and 11% had atrial fibrillation, while only 4% had a history of cerebrovascular disease (Table 1).

**Table 1:** Demographic and clinical characteristics of patients (n=45).

General details		Number of patients (n=45)	Percentage (%)
Age group (in years) (mean±SD=48±14.1)	21-30	5	11%
	31-40	10	22%
	41-50	1	2%
	51-60	22	50%
	61-70	6	13%
	81-90	1	2%
Gender	Male	24	53%
	Female	21	47%
Comorbidities	Diabetes mellitus	25	56%
	Hypertension	29	64%
	Ischemic heart disease	13	29%
	Atrial fibrillation	5	11%
	Old cerebrovascular disease	2	4%
	Smoking	15	33%

The atherosclerotic disease was diagnosed in 67% of cases, followed by CNS vasculitis (22%) and moyamoya disease (11%). VW-MRI revealed non-vulnerable atherosclerosis in 20 patients, vulnerable atherosclerosis in 10, CNS vasculitis in 10, and moyamoya disease in five (Table 2).

**Table 2:** Distribution of disease in patients. CNS: central nervous system

Distribution of diseases	Number (n)	Percentage (%)	P-value
Atherosclerotic disease	30	67%	0.003
CNS vasculitis	10	22%	0.347
Moyamoya disease	5	11%	0.022
Mean±SD	15±13.23		

Characteristics of intracranial atherosclerotic disease, vasculitis, and moyamoya disease are highlighted in Table 3, Table 4, and Table 5. We found severe luminal narrowing in 47% of cases and eccentric plaques in 53%, consistent with Mazzacane et al. [4] and Kim et al. [7]. For CNS vasculitis, VW thickening and positive remodeling align with Farag et al. [5] and Adhithyan et al. [8]. In moyamoya, negative remodeling and arterial narrowing mirror Mandell et al.'s findings. MRA detected significant intracranial stenosis (>50%) in 47% of atherosclerotic patients (chi-square=9.421 with 2 degrees of freedom; p=0.009) and 11% of moyamoya cases. VW-MRI provided better differentiation, identifying pathologies in all of the cases as compared to MRA-identified pathology in 73.3% of cases. Vascular remodeling was present in 16 cases, with positive remodeling observed in five patients with CNS vasculitis and six with vulnerable atherosclerosis, while moyamoya disease had five cases with negative remodeling. Positive remodeling will lead to a higher plaque burden and increased stroke rate [9].

**Table 3:** Summarized VW-MRI findings in cases of intracranial atherosclerotic disease. VW-MRI: vessel wall magnetic resonance imaging; ICA: internal carotid artery; MCA: middle cerebral artery; ACA: anterior cerebral artery

Characteristics	Details
Luminal compromise	Present
VW thickening	Eccentric and irregular
Enhancement	Present
Signal intensity of various plaque components	Cholesterol: T1 isointense/T2 hypointense; fibrous plaque: T1/T2 isointense; calcification: T1/T2 dark; fibrous cap: juxtaluminal T2 hyperintense
Distribution	Mostly distal ICA and proximal MCA and ACA

**Table 4:** Summarized VW-MRI findings in cases of CNS vasculitis. VW-MRI: vessel wall magnetic resonance imaging; CNS: central nervous system

Characteristics	Details
Luminal compromise	Present
VW thickening	Concentric
Enhancement	Present
Signal intensity	Homogeneous isointense T2 signal intensity
Distribution	Multifocal involving medium and small blood vessels

**Table 5:** Summarized VW-MRI features in cases of moyamoya disease. VW-MRI: vessel wall magnetic resonance imaging; ICA: internal carotid artery; MCA: middle cerebral artery

Characteristics	Details
Luminal compromise	Progressive stenosis of ICA and proximal MCA negative remodeling
VW thickening	Thickened/normal
Enhancement	Absent
Signal intensity	Homogeneous isointense T2 signal intensity
Distribution	Distal ICA and proximal MCA

**Figure 1:**
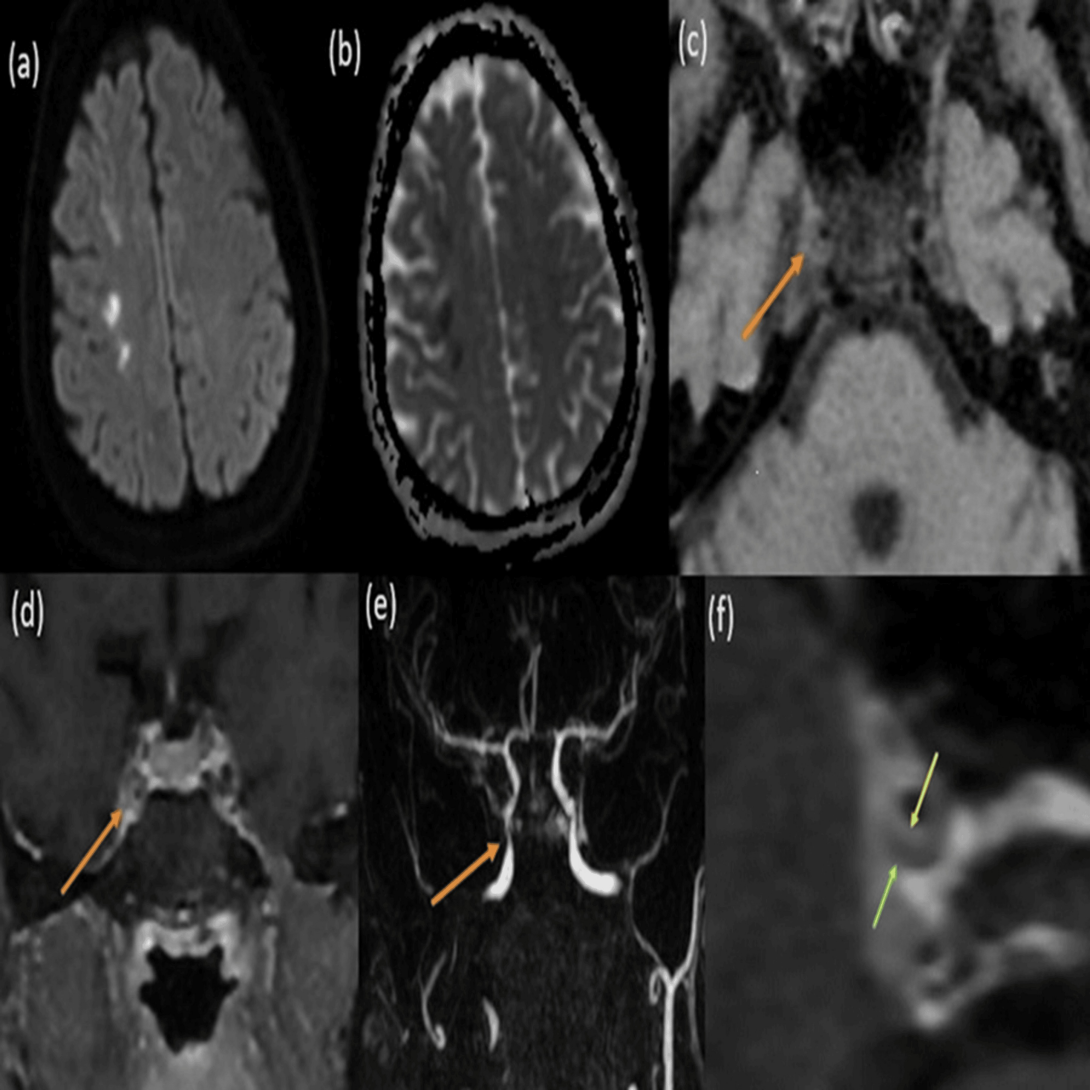
Intracranial atherosclerosis: (a and b) Axial DWI/ADC images demonstrating acute infarcts in the right subcortical and deep white matter of the right frontal lobe. (c and d) VW-MRI axial T1 BB and coronal T1 BB +C sequences demonstrating isointense eccentric wall thickening and grade 2 enhancement (orange arrows) involving a cavernous segment of the right ICA causing moderate stenosis demonstrated in CE-MRI sequence in (e) (orange arrow). (f) Magnified image of the site of a plaque showing a contrast-enhancing fibrous cap (yellow arrow) and non-enhancing plaque in the image (green arrow). DWI/ADC: diffusion-weighted imaging/apparent diffusion coefficient; VW-MRI: vessel wall magnetic resonance imaging; BB: black blood; ICA: internal carotid artery; CE-MRI: contrast-enhanced magnetic resonance imaging

VW-MRI can reveal distinct characteristics that help to differentiate between intracranial atherosclerosis, CNS vasculitis, and moyamoya disease. In cases of intracranial atherosclerosis, the imaging typically shows arterial wall thickening with an irregular inner margin and eccentric (non-uniform) enhancement as depicted in Figure 1.

**Figure 2:**
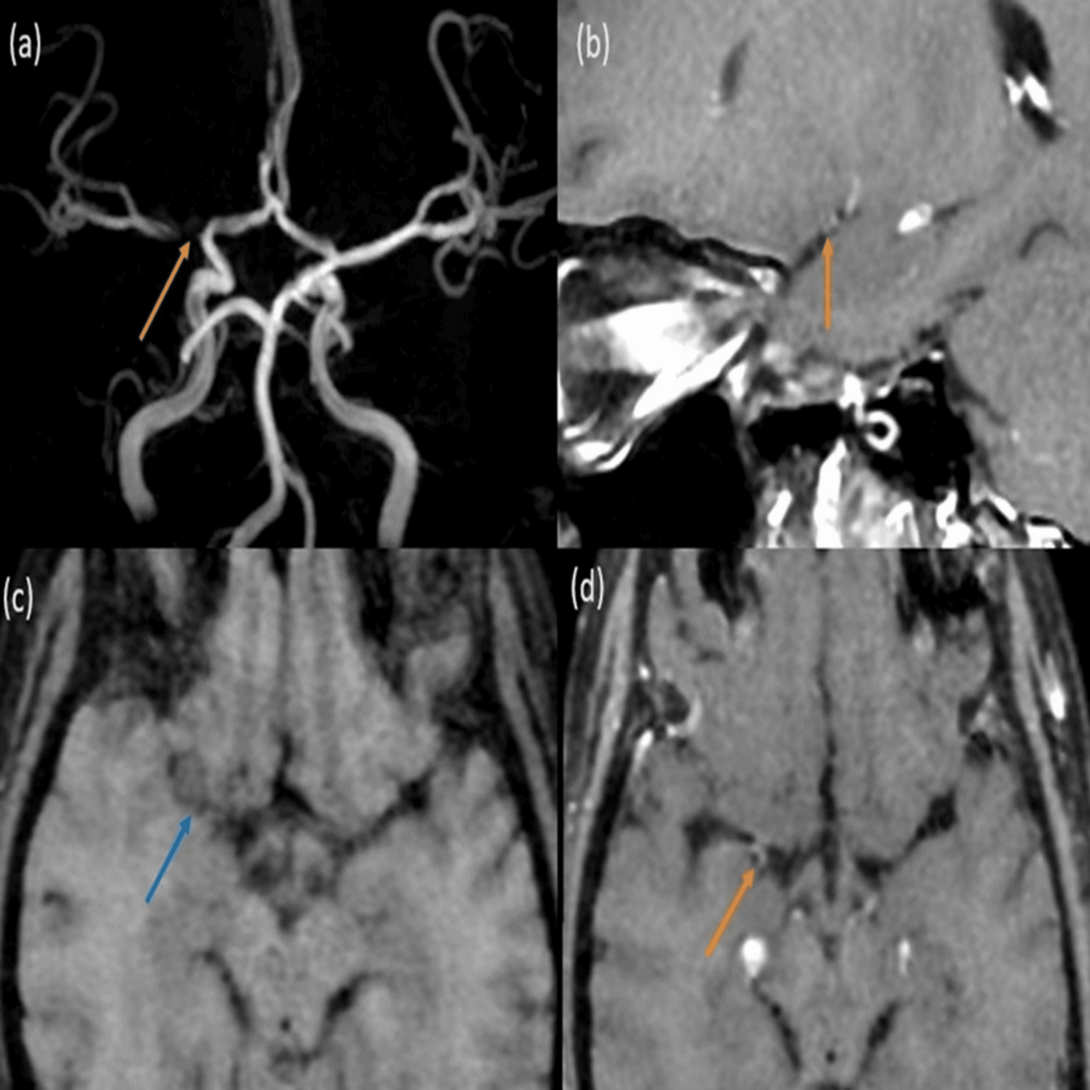
CNS vasculitis: (a) TOF MRA images demonstrating occlusion of the right proximal M1 MCA segment (orange arrow). (b and d) VW-MRI sagittal and post-contrast axial T1 BB sequences demonstrating circumferential irregular wall thickening and enhancement (orange arrows) involving the right proximal M1 MCA segment with its complete occlusion. (c) There is also the presence of non-enhancing dirty CSF in the right Sylvian cistern likely due to basal exudates (blue arrow). Diagnosis of granulomatous tuberculous vasculitis is considered. CNS: central nervous system; TOF MRA: time-of-flight magnetic resonance angiography; VW-MRI: vessel wall magnetic resonance imaging; BB: black blood; MCA: middle cerebral artery; CSF: cerebrospinal fluid

In CNS vasculitis, VW-MRI generally shows circumferential (uniform) enhancement of the arterial wall with a smooth, regular inner margin as shown in Figure 2. This pattern is indicative of inflammation and is distinctly different from the irregular and non-uniform enhancement seen in atherosclerosis.

**Figure 3:**
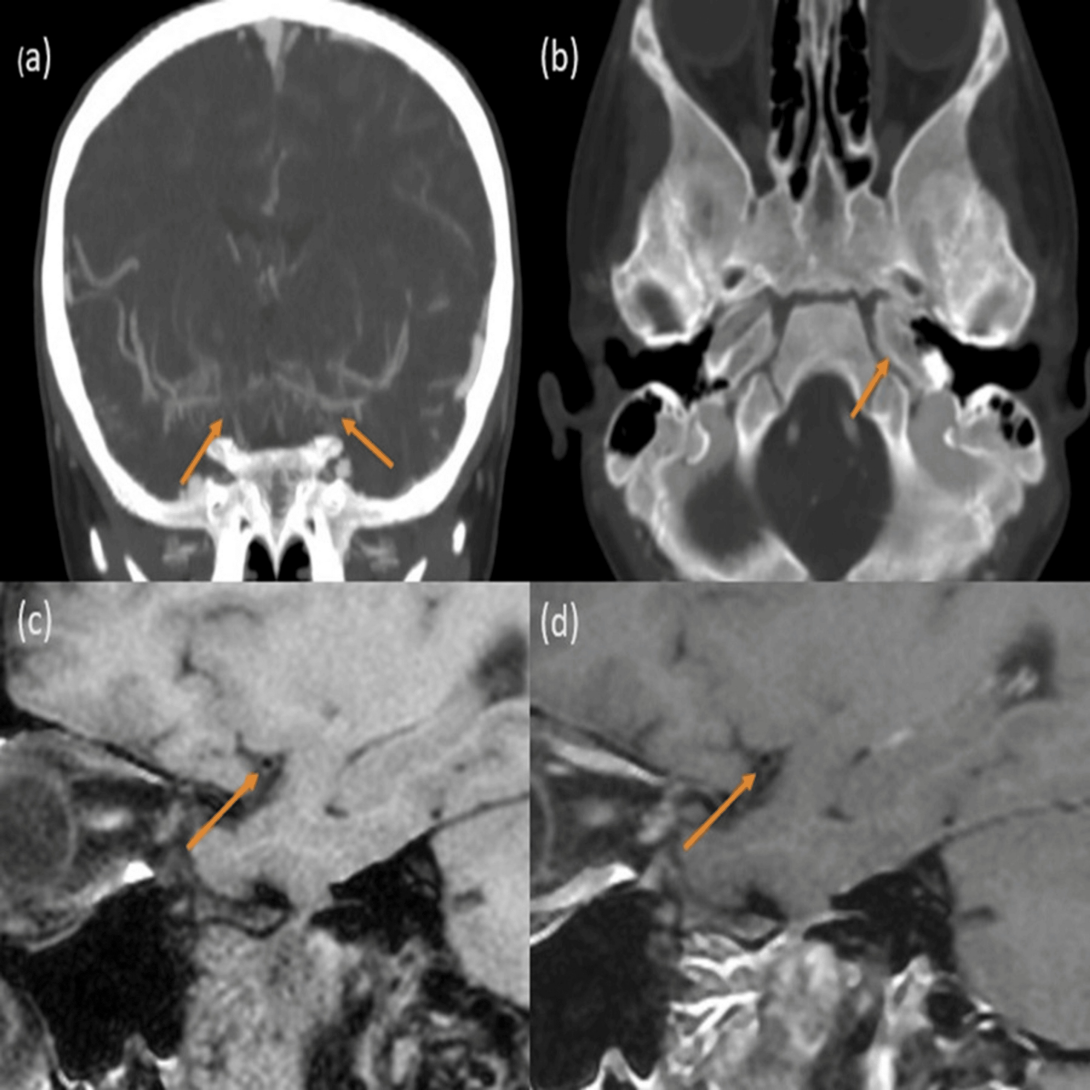
Moyamoya disease: (a) Coronal CTA images demonstrating complete occlusion of the right terminal ICA, A1 ACA, and the proximal M1 MCA and non-opacification of the left terminal ICA (agenesis) (orange arrows) with chronically developed collaterals (right>left). (b) CECT head image (bone window - agenesis of the left ICA is noted). (c and d) VW-MRI sagittal T1 BB pre- and post-contrast images demonstrating isointense circumferential non-enhancing wall thickening of the right M1 MCA (orange arrows). Diagnosis of moyamoya disease with agenesis of the left ICA is considered. CTA: computed tomography angiography; ICA: internal carotid artery; ACA: anterior cerebral artery; MCA: middle cerebral artery; VW-MRI: vessel wall magnetic resonance imaging; BB: black blood; CECT: contrast-enhanced computed tomography

Meanwhile, in moyamoya disease, the imaging reveals a narrowing of the luminal artery without any post-contrast enhancement as depicted in Figure 3.

## Discussion

The demographic analysis of our study population including 45 patients reveals significant insights into the age and gender distribution among patients with cerebrovascular conditions. The majority of the patients (50%) fall within the 51-60-year age group, suggesting that cerebrovascular diseases predominantly affect middle-aged individuals. This age group is followed by the 31-40-year age group (22%), highlighting a considerable presence of cerebrovascular conditions in relatively younger individuals as well. The study population exhibits a high prevalence of comorbidities, with hypertension being the most common (64%). The presence of smoking in 33% of the population highlights the role of lifestyle factors in the development of these conditions. The primary objective of our study was to assess suspicious lesions detected by MRA for further evaluation by VW-MRI. The analysis of disease distribution shows that atherosclerotic disease is the most prevalent (67%), followed by CNS vasculitis (22%) and moyamoya disease/pattern (11%). VW-MRI can reveal distinct characteristics that help differentiate between intracranial atherosclerosis, CNS vasculitis, and moyamoya disease. In cases of intracranial atherosclerosis, the imaging typically shows arterial wall thickening with an irregular inner margin and eccentric (non-uniform) enhancement. These features are often associated with a narrowed luminal artery and, in some instances, a hemorrhagic component, indicating that the atherosclerotic plaque is active or vulnerable. Conversely, if the narrowed artery and wall thickening are present but there is no enhancement or hemorrhagic component, the plaque is considered inactive or stable. In CNS vasculitis, VW-MRI generally shows circumferential (uniform) enhancement of the arterial wall with a smooth, regular inner margin. This pattern is indicative of inflammation and is distinctly different from the irregular and non-uniform enhancement seen in atherosclerosis. Meanwhile, in moyamoya disease, the imaging reveals a narrowing of the luminal artery without any post-contrast enhancement. Additionally, there are no signs of hemorrhagic content or fatty deposits within the VW, highlighting a characteristic feature of moyamoya, where the narrowing occurs without inflammatory or hemorrhagic changes. Our results are also in agreement with the literature reported by Mandell and colleagues in 2017 who found that VW-MRI of intracranial atherosclerotic plaque typically demonstrates arterial wall thickening, which eccentrically (non-uniformly) involves the circumference of the arterial wall, whereas VW-MRI demonstrates smooth, homogeneous, concentric arterial wall thickening and enhancement in patients with CNS vasculitis [10]. Also as regards vasculitis, similar findings were reported by Mandell and colleagues in 2012 who used VW-MRI to differentiate between RCVS and CNS vasculitis [11]. Also as regards plaque activity (vulnerability), nearly similar findings were reported by Vergouwen and colleagues in 2011. In his study, eight patients were identified to characterize the VW-MRI findings and enhancement patterns in the middle cerebral artery of patients with presumed atherosclerotic disease and recent infarction in the territory of the affected artery using 3-T MRI [12]. Plaque enhancement on T1-weighted post-contrast sequences is a characteristic of symptomatic plaque and reflects intralesional inflammation and neovascularization [4]. This is a hallmark of a causal relationship between the plaque and the ischemic event, and the more intense the plaque enhancement, the greater the probability of such an association [5]. Also, the presence of hemorrhage in the plaques, which appear as T1-weighted hyperintense, is associated with an increased risk of stroke [6]. On the other hand, vasculitis had concentric thickening and concentric contrast enhancement and in some cases the presence of positive remodeling. Our study also helped to identify the features which can depict plaque vulnerability in the intracranial atherosclerotic disease subgroup in 10 patients which include these features like narrowed artery lumen with wall thickening with irregular inner margin, eccentric enhancement, and hemorrhagic component. The comparison between MRA and VW-MRI demonstrates the superior ability of VW-MRI to detect VW pathology. While MRA identified pathology in 73.3% of cases, VW-MRI detected it in 100% of cases, highlighting its efficacy in diagnosing cerebrovascular conditions. In a study done by Swartz et al., the diagnostic accuracy of etiology increased from 43.5% after luminal imaging to 96.3% when luminal high-resolution VW-MRI (HRVW-MRI) were reviewed [13]. 

## Conclusions

We currently use traditional methods like MRA/CTA/DSA to evaluate intracranial vasculopathies. However, these methods have limited specificity for disease differentiation. This is especially problematic in cases of CNS vasculitis and RCVS where different treatment options are necessary. On the other hand, VW-MRI is a high-resolution imaging technique that allows for the visualization of VW rather than just luminal narrowing. This helps us to better characterize intracranial vasculopathies like atherosclerosis, vasculitis, moyamoya, dissection, and RCVS. Additionally, VW-MRI can also play a role in atherosclerotic plaque characterization. The need for high-resolution imaging of small, tortuous intracranial arteries, therefore VW-MRI, can be time-consuming and may lead to motion artifacts, which could limit its use.
